# A fluorescent reporter system for tracking *Lactobacillus casei* T1 in the murine gastrointestinal tract

**DOI:** 10.3389/fmicb.2026.1892263

**Published:** 2026-08-27

**Authors:** Dengxiong Hua, Xuexue Zhou, Ji Lan, Daoyan Wu, Yingqian Kang, Zhihao Yu, Zhengrong Zhang, Yina Yao, Wei Hong, Zhenghong Chen, Guzhen Cui

**Affiliations:** 1 Guizhou Key Laboratory of Microbiome and Infectious Disease Prevention and Control, Clinical Laboratory Center, Affiliated Hospital of Guizhou Medical University, Guiyang, Guizhou, China; 2 Key Laboratory of Ministry of Education for Endemic and Ethnic Diseases and Key Laboratory of Medical Molecular Biology of Guizhou, Guizhou Medical University, Guiyang, Guizhou, China

**Keywords:** fluorescent reporter system, *in vivo* imaging, *Lactobacillus casei* T1, mKate, probiotic tracking, promoter optimization

## Abstract

**Background:**

Fluorescent reporter systems are useful for studying probiotic colonization and host–microbe interactions. However, their use in lactic acid bacteria is still limited by relatively weak fluorescence signals, insufficient expression stability, and limited resolution during in vivo imaging. In particular, efficient strain-specific tracking systems remain scarce.

**Methods:**

Here, we developed a red fluorescent reporter system for *Lactobacillus casei* T1 (*L.c* T1). Lactate dehydrogenase (LDH) promoters identified from the *L.c* T1 genome were compared with the constitutive P32 promoter to drive expression of the red fluorescent proteins mCherry and mKate. The different promoter–reporter combinations were evaluated in both *Escherichia coli* DH5α and *L.c* T1. Fluorescence expression was further examined under different environmental pH conditions. The optimized reporter strains were then evaluated by whole-body fluorescence imaging in living mice and ex vivo imaging of gastrointestinal tissues following oral administration.

**Results:**

Among the constructs tested, P32-mKate produced the strongest and most stable fluorescence signal in *L.c* T1. Fluorescence intensity was influenced by environmental pH, with higher signals observed under mildly alkaline conditions. Whole-body fluorescence imaging showed that the engineered strain could be detected in living mice following oral administration. Ex vivo imaging of gastrointestinal tissues provided clearer localization of fluorescence, with signals mainly detected in the stomach and upper small intestine.

**Conclusion:**

We established a stable and efficient red fluorescent reporter system for *L.c* T1. The P32-mKate system enables detection of the engineered strain both in vitro and in vivo and provides a practical approach for tracking probiotic distribution and studying host–microbe interactions in preclinical animal models.

## 1 Introduction

Probiotics play important roles in maintaining intestinal homeostasis, modulating host immune responses, and alleviating metabolic disorders (Xu et al., 2026). In recent years, they have been widely applied in functional foods, biotherapeutics, and engineered living systems (Wang and Zhong, 2024). With the rapid development of synthetic biology, lactic acid bacteria (LAB) are not only used as vehicles for delivering bioactive molecules but are also being increasingly engineered as programmable living therapeutics responsive to the intestinal microenvironment (Fan et al., 2024; Guo et al., 2024; Li et al., 2024). However, investigations of probiotic colonization dynamics, gastrointestinal transit, and *in vivo* delivery efficiency critically depend on robust, sensitive, and non-invasive tracking systems (George et al., 2018; Yang et al., 2019). Therefore, the development of efficient fluorescence imaging platforms for monitoring lactic acid bacteria in preclinical animal models has become a key technical requirement in probiotic research.

Fluorescent reporter systems have been widely employed to investigate bacterial colonization and host–microbe interactions because they enable sensitive, non-destructive, and longitudinal fluorescence monitoring (Dib et al., 2025; Korwin-Mihavics et al., 2025; Zhai et al., 2025). Green fluorescent protein (GFP) and its derivatives have been successfully applied in several model bacterial systems for fluorescence-based tracking in animal models (Tian et al., 2024; Wu et al., 2025). However, their application in lactic acid bacteria remains limited. On the one hand, the genetic manipulation systems of lactic acid bacteria are relatively less mature, and substantial variability in promoter compatibility and heterologous protein expression efficiency among strains often leads to unstable fluorescence signals (Yang et al., 2021). On the other hand, strong tissue autofluorescence in the gastrointestinal tract reduces the signal-to-noise ratio of GFP-based imaging systems *in vivo*. In contrast, red fluorescent proteins, with longer excitation and emission wavelengths and improved tissue penetration, are more suitable for gastrointestinal imaging applications (Wang et al., 2020; Xiong et al., 2025). Nevertheless, systematic studies on red fluorescent expression systems optimized for lactic acid bacteria, particularly *Lactobacillus casei*, remain scarce, and stable, high-efficiency reporter systems for fluorescence-based monitoring in animal models have yet to be established.

Promoters are key determinants of heterologous gene expression levels. The constitutive P32 promoter, commonly used in the pMG36E expression vector, has been widely applied for heterologous protein expression in lactic acid bacteria due to its relatively strong constitutive activity (Ni et al., 2017). However, transcriptional regulation varies significantly among different *Lactobacillus* hosts, and endogenous promoters derived from the host genome may offer improved expression stability and compatibility (Van Zyl et al., 2018). In particular, promoters associated with central metabolic pathways, such as those of lactate dehydrogenase (LDH), may provide enhanced adaptability for heterologous gene expression (Ganguly et al., 2020). In addition, red fluorescent proteins differ in maturation efficiency, photostability, and environmental sensitivity, highlighting the need for systematic evaluation of promoter-reporter combinations to achieve optimal performance.

*Lactobacillus casei* T1 (*L.c* T1), previously isolated from Tibetan yak milk in our laboratory, has been shown to possess favorable intestinal adaptability and probiotic potential (Luo et al., 2011; Wu et al., 2021; Yu et al., 2023). However, a suitable imaging system for *in vivo* visualization of this strain is still lacking, which limits further investigation of its colonization behavior and host interaction mechanisms. In this study, we used *L.c* T1 as the model strain to predict and screen endogenous LDH promoters and constructed red fluorescent expression systems using mKate and mCherry under different promoter controls. The expression efficiency and stability of these systems were systematically evaluated in both *Escherichia coli* DH5α and *L.c* T1. Furthermore, fluorescence expression conditions were optimized, and whole-body fluorescence imaging of living mice, together with ex vivo imaging of gastrointestinal tissues, was performed to evaluate the gastrointestinal distribution of the engineered strain. This study aims to establish a red fluorescent reporter platform for non-invasive fluorescence monitoring of *L.c* T1 in preclinical animal models, providing a technical foundation for studies of probiotic colonization, host–microbe interactions, and the development of engineered probiotic systems.

## 2 Results

### 2.1 Promoter prediction and fluorescent reporter vectors construction

**Figure 1:**
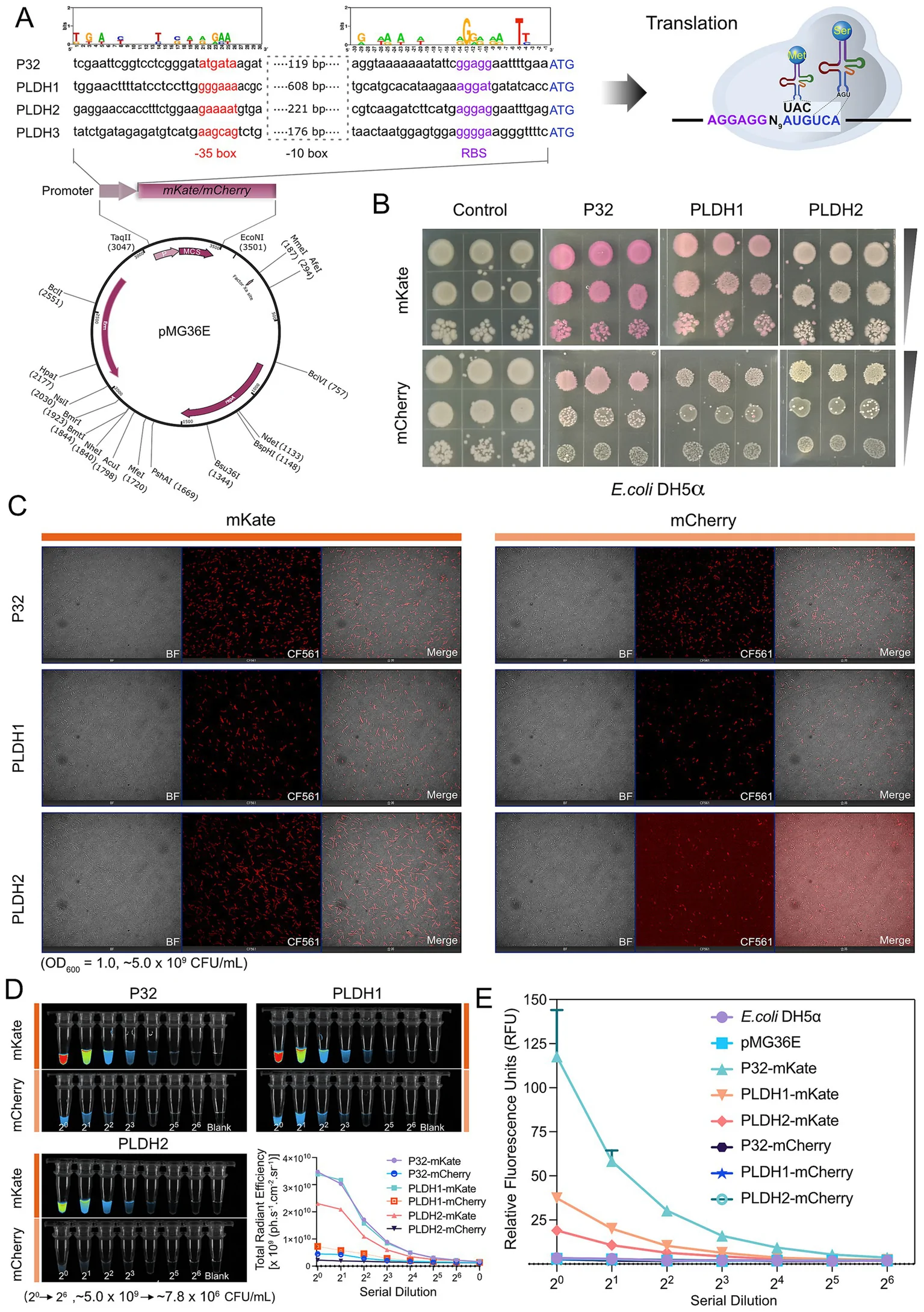
Prediction of promoter sequences and construction of fluorescent expression systems in *E. coli* DH5α. **(A)** Prediction of putative LDH promoter sequences from the *L.c T1* genome. **(B)** Fluorescence phenotype of engineered *E. coli* DH5α strains grown on LB agar plates. **(C)** Confocal laser scanning microscopy analysis of fluorescence expression in recombinant *E. coli* DH5α strains. **(D)** In vivo imaging system-based detection of fluorescence intensity in serially diluted *E. coli* DH5α strains. **(E)** Quantitative analysis of fluorescence intensity under different dilution gradients.

To construct an efficient fluorescent expression system, the plasmid pMG36E was selected as the backbone for promoter optimization. The well-characterized P32 promoter within pMG36E, along with two previously reported *Lactobacillus* promoters (P23 and POL2) (Meng et al., 2021), were used as references. Multiple sequence alignment was performed using ClustalW to identify conserved promoter functional elements, including the −35 region (5′-ATGACA-3′), the −10 region (5′-TAAAAT-3′), and the ribosome binding site (RBS, 5′-GGGAGG-3′) (Figure 1A). Based on conserved sequence features of these functional elements, three putative promoter sequences were predicted from the genome of *Lactobacillus casei* T1 within the lactate dehydrogenase (LDH) gene region (designated PLDH1, PLDH2, and PLDH3). Subsequently, the red fluorescent protein genes *mKate* and *mCherry* were selected as reporter genes. Recombinant plasmids pMG36E-Px-mKate and pMG36E-Px-mCherry (where X represents P32, PLDH1, or PLDH2) were constructed accordingly. The transcriptional activity of different promoter–fluorescent protein combinations was systematically evaluated in both *Escherichia coli* DH5α and *L.c* T1, aiming to establish a robust fluorescent expression system capable of efficient performance in both heterologous and native *Lactobacillus* hosts.

### 2.2 Evaluation of fluorescent protein expression in *E.Coli* DH5α

To identify an efficient fluorescent expression system, recombinant plasmids pMG36E-Px-mKate and pMG36E-Px-mCherry were transformed into *Escherichia coli* DH5α. After transformation, colonies harboring P32-driven mKate or mCherry constructs exhibited a distinct red coloration on selective agar plates (Figure 1B). Confocal laser scanning microscopy further confirmed that all constructs produced detectable fluorescence signals; however, the Px-mKate systems showed markedly stronger fluorescence compared with the Px-mCherry counterparts (Figure 1C). Quantitative analysis of fluorescence intensity consistently demonstrated significantly higher signal levels in the Px-mKate group. Among all tested combinations, the P32-mKate construct exhibited the strongest fluorescence performance (Figures 1D,E).

### 2.3 Evaluation of fluorescent protein expression in *L.c* T1

**Figure 2:**
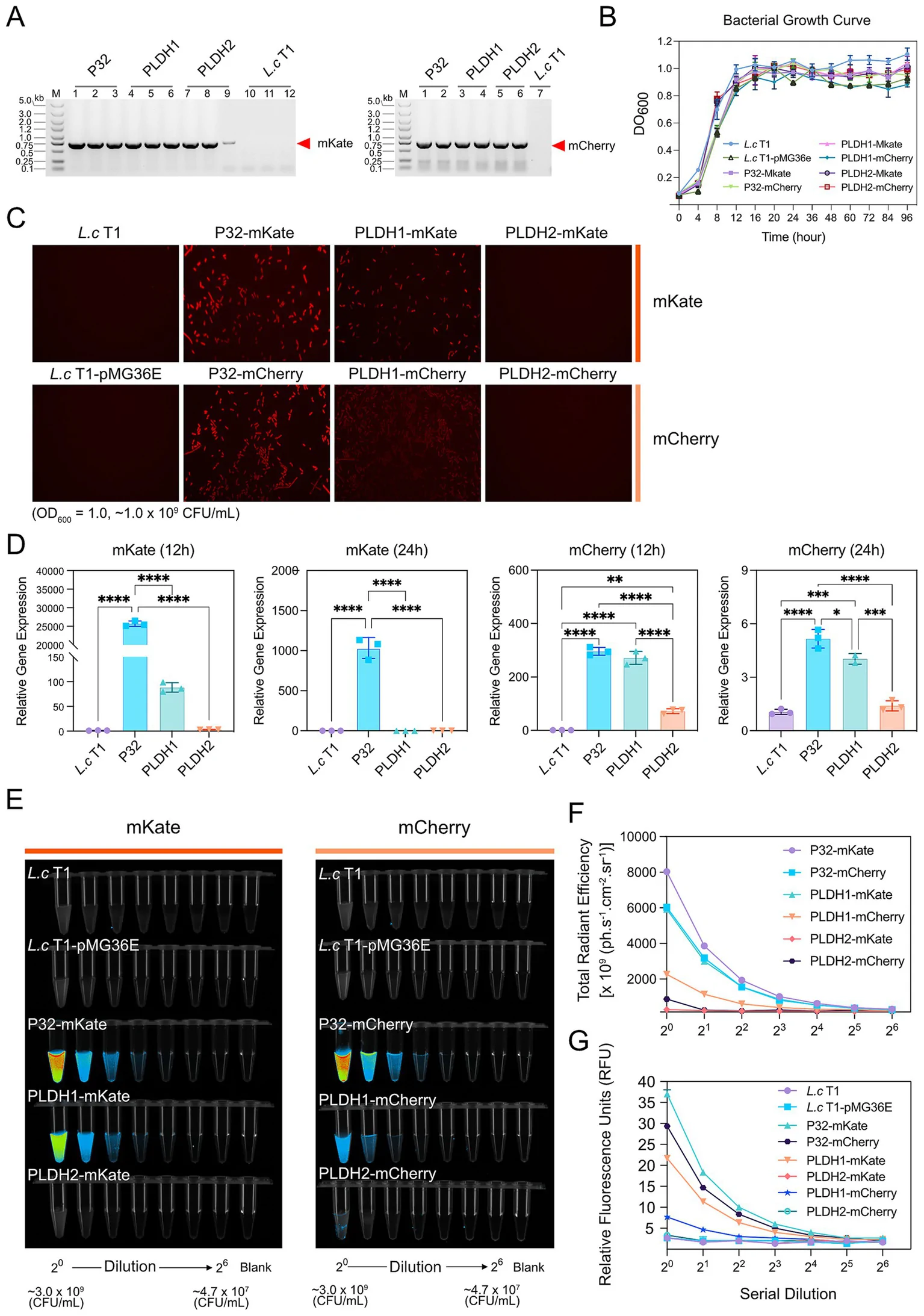
Construction and fluorescence characterization of engineered *L.c* T1 strains. **(A)** PCR confirmation of fluorescent plasmid electrotransformants in *L.c* T1. Left: Lanes 1–3, P32-mKate; 4–6, PLDH1-mKate; 7–9, PLDH2-mKate; 10–12, *L.c* T1. Right: Lanes 1–2, P32-mCherry; 3–4, PLDH1-mCherry; 5–6, PLDH2-mCherry; 7, *L.c* T1. **(B)** Growth curves of wild-type, empty vector control, and engineered *L.c* T1 strains. **(C)** Representative confocal laser scanning microscopy images of fluorescence expression in *L.c* T1 strains. **(D)** qPCR analysis of fluorescent gene expression in *L.c* T1-P32-mKate and *L.c* T1-P32-mCherry at 12 h and 24 h of culture. **(E,F)** In vivo imaging system-based detection of fluorescence intensity in serially diluted *L.c* T1 strains. **(G)** Fluorescence detection and quantitative analysis of engineered strains using a microplate reader under different dilution gradients.

To evaluate the performance of the constructed fluorescent expression systems in *L.c* T1, recombinant plasmids pMG36E-Px-mKate and pMG36E-Px-mCherry were further transformed into *L.c* T1. Transformants were verified by detecting the mKate and mCherry coding sequences in the recombinant strains. A total of six fluorescently labeled *L.c* T1 strains were successfully obtained, representing combinations of three promoters (P32, PLDH1, and PLDH2) and two fluorescent proteins (mKate and mCherry) (Figure 2A).

To assess whether heterologous expression affected bacterial fitness, growth curves of all engineered strains were determined. No significant differences in growth were observed between fluorescent strains, wild-type *L.c* T1, and empty vector controls, indicating that introduction of the expression system did not impose a measurable metabolic burden (Figure 2B). Fluorescence expression was then examined using confocal laser scanning microscopy and quantitative PCR. Detectable fluorescence signals were observed in strains harboring P32-mKate, P32-mCherry, PLDH1-mKate, and PLDH1-mCherry constructs, whereas PLDH2-driven strains exhibited relatively weak fluorescence signals. Among all promoter–reporter combinations, constructs driven by the P32 promoter showed the strongest fluorescence intensity, outperforming both PLDH1 and PLDH2 systems (Figures 2C,D).

Consistently, quantitative fluorescence analysis confirmed that the P32-mKate strain exhibited significantly higher fluorescence intensity than all other engineered strains, demonstrating the best overall performance among all tested promoter–fluorescent protein combinations (Figures 2E–G).

### 2.4 Optimizing expression conditions for the *L.c* T1-P32-mKate

**Figure 3:**
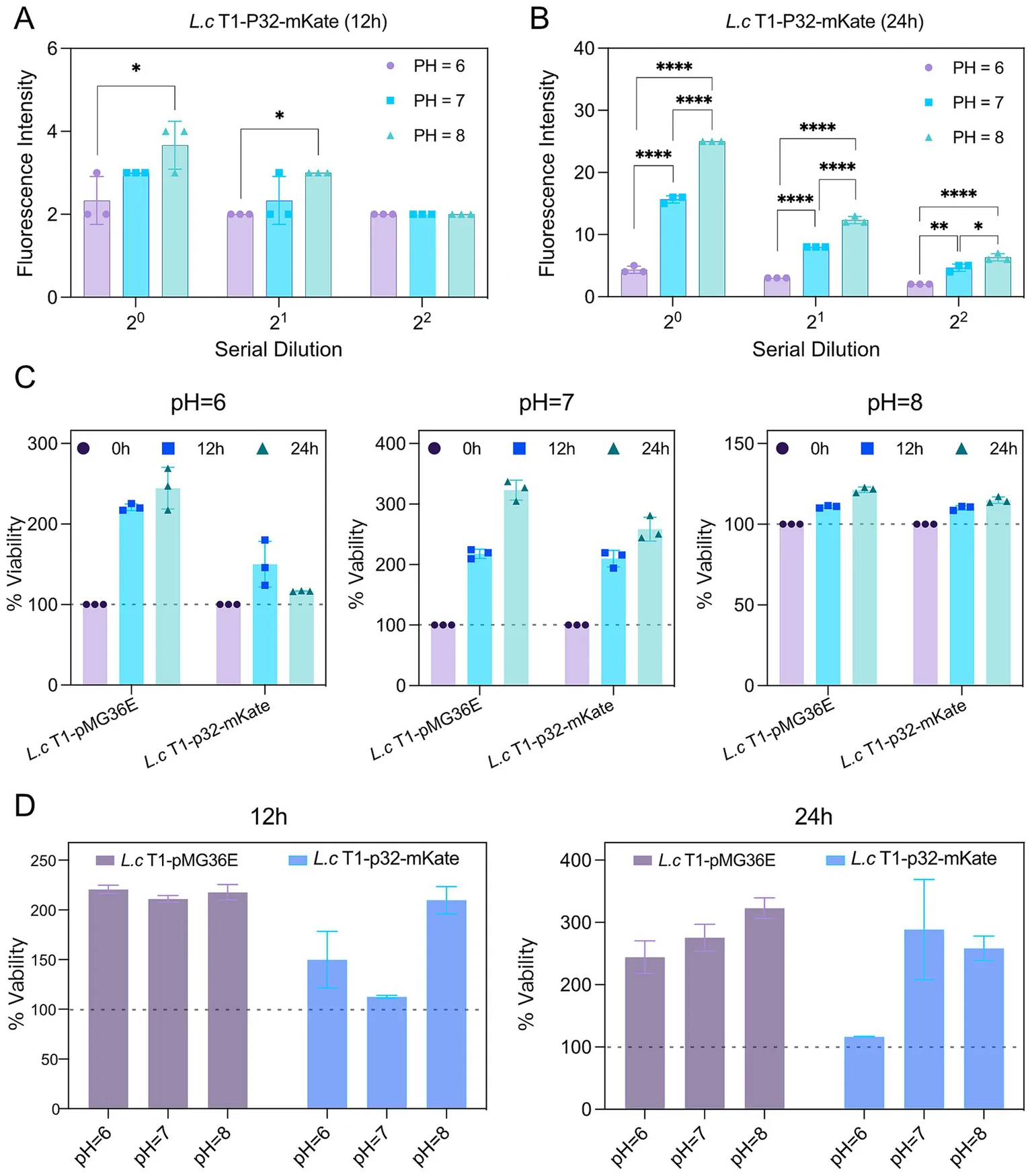
Optimization of fluorescence expression conditions for *L.c* T1-P32-mKate. **(A,B)** Effect of environmental pH on the fluorescence intensity of *L.c* T1-P32-mKate after 12 h and 24 h of incubation. **(C,D)** Survival rates of *L.c* T1-P32-mKate after incubation under different pH conditions for 12 h and 24 h.

Gastrointestinal pH is an important indicator of acid–base homeostasis within the intestinal microenvironment, which is generally maintained within a physiological range of 5.5–7.0. To evaluate the effect of environmental pH on fluorescence expression of *L.c* T1-P32-mKate, fluorescence intensity was measured after 12 h and 24 h of incubation under different pH conditions (6.0, 7.0, and 8.0) and at different bacterial dilution levels (2^0^, 2^1^, and 2^2^). As shown in Figure 3A, fluorescence expression of *L.c* T1-P32-mKate exhibited a clear pH-dependent pattern. After 12 h of incubation, overall fluorescence signals remained relatively low, with statistically significant differences observed only between pH 8.0 and pH 6.0 under low dilution conditions (2^0^ and 2^1^). Upon extending incubation to 24 h, fluorescence intensity increased markedly, and the regulatory effect of pH became more pronounced. Across all dilution levels, fluorescence intensity increased progressively with rising pH (from 6.0 to 8.0), showing a clear stepwise enhancement. At the undiluted level (2^0^), fluorescence intensity at pH 8.0 was approximately six-fold higher than that at pH 6.0. Even at higher dilution (2^2^), fluorescence signals under pH 8.0 remained significantly higher than those observed under acidic conditions (Figure 3B). Viable cell counts showed that the survival rate of *L.c* T1-P32-mKate exceeded 100% under all tested pH conditions (Figures 3C,D), indicating that the observed differences in fluorescence were not associated with reduced bacterial viability. Taken together, these results demonstrate that the P32-driven mKate expression system in *L.c* T1 performs more efficiently under mildly alkaline conditions, and that this enhancement becomes more evident with prolonged incubation and is maintained across different bacterial dilution levels.

### 2.5 Fluorescence-based visualization of engineered *L.c* T1 in the mouse gastrointestinal tract

**Figure 4:**
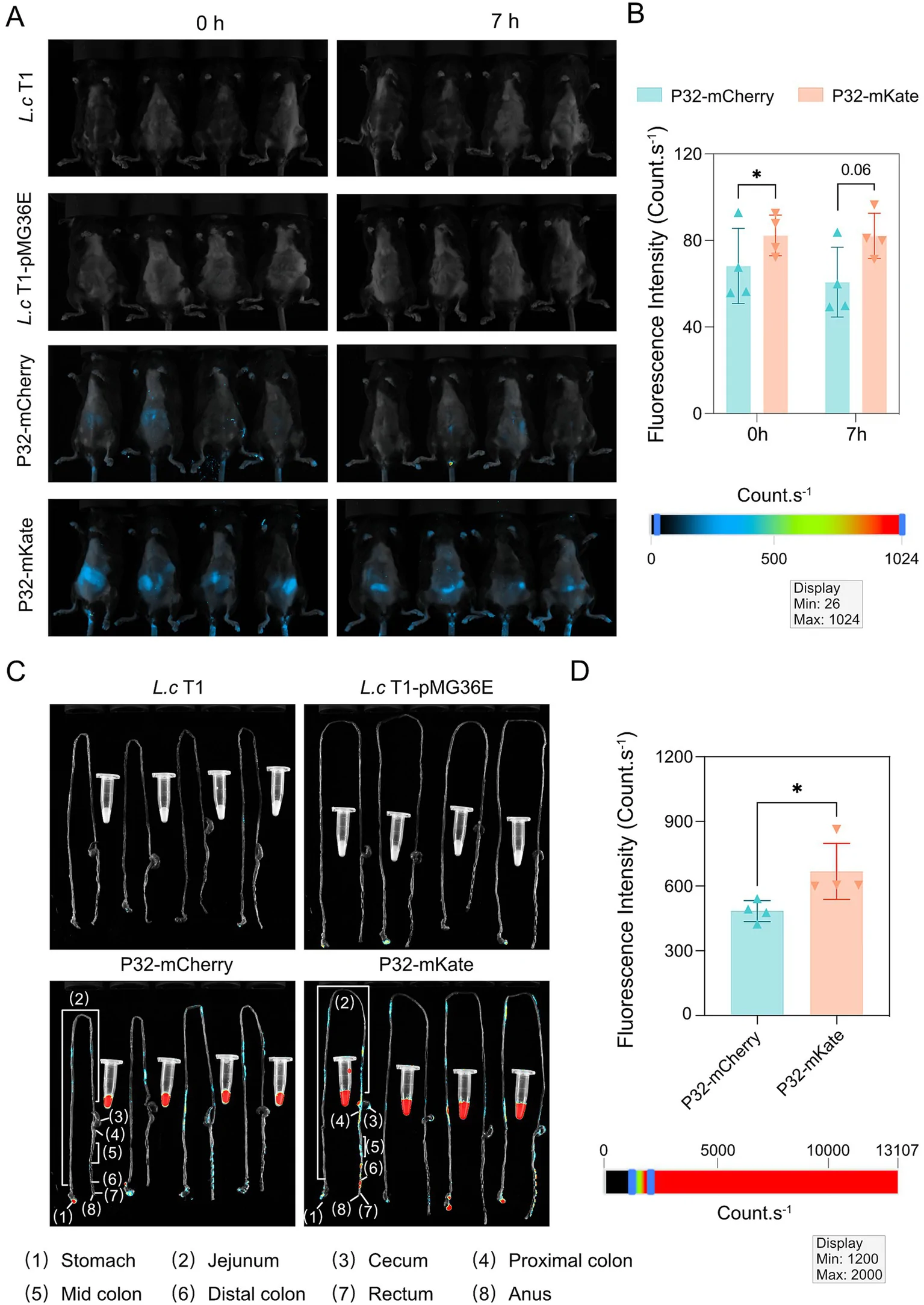
Non-invasive fluorescence imaging and gastrointestinal localization of engineered *L.c.* T1 in mice. **(A)** Whole-body fluorescence imaging of living mice after oral administration of engineered *L.c.* T1 strains and **(B)** The corresponding quantification of average fluorescence intensity. **(C)** Ex vivo fluorescence imaging of dissected gastrointestinal tracts at 7 h post-gavage, showing bacterial distribution and **(D)** The quantitative analysis of mean fluorescence intensity within defined regions of interest (ROIs).

To evaluate the feasibility of fluorescence-based visualization of engineered *L.c* T1 *in vivo*, mice were orally administered with *L.c* T1-P32-mKate, *L.c* T1-P32-mCherry, wild-type *L.c* T1, or the empty vector control strain. Whole-body fluorescence imaging was performed at 0 h and 7 h post-gavage using an in vivo imaging system (Figure 4A). Detectable red fluorescence signals were observed in the abdominal regions of mice receiving *L.c* T1-P32-mKate or *L.c* T1-P32-mCherry, while no obvious signals were detected in the control groups (Figure 4A). Quantitative analysis of fluorescence intensity showed that *L.c* T1-P32-mKate produced significantly stronger signals than *L.c* T1-P32-mCherry at 0 h after administration (*p* < 0.05). Although the fluorescence intensity of *L.c* T1-P32-mKate remained higher at 7 h, the difference compared with *L.c* T1-P32-mCherry was not statistically significant (*p* = 0.06) (Figure 4B).

To further assess bacterial distribution within the gastrointestinal tract, excised gastrointestinal tissues were subjected to ex vivo fluorescence imaging. As shown in Figure 4C, both fluorescent strains exhibited clear red fluorescence signals, mainly localized in the stomach and intestinal regions. Quantitative analysis (Figure 4D) revealed that the fluorescence intensity of the *L.c* T1-P32-mKate group was significantly higher than that of the *L.c* T1-P32-mCherry group under the same detection conditions. Together, these results demonstrate that both P32-driven mKate and mCherry systems enable fluorescence visualization of engineered *L.c* T1 in mice, with the P32-mKate system exhibiting stronger fluorescence intensity under the conditions tested.

## 3 Discussion

The development of fluorescence-based tracking approaches for lactic acid bacteria is important for investigating probiotic colonization dynamics, spatial distribution, and functional activity within the host. However, the application of such systems remains limited by immature genetic manipulation tools, insufficient stability of heterologous protein expression, and strong autofluorescence background in the gastrointestinal tract (Van Zyl et al., 2018). This system enabled non-invasive monitoring of engineered bacteria in living mice and further allowed ex vivo visualization of their distribution along the gastrointestinal tract.

Promoter selection is a key determinant of heterologous gene expression efficiency (Deal et al., 2024; Sun et al., 2025). In addition to the well-characterized constitutive promoter P32, several putative LDH promoters were predicted and identified from the genome of *L.c* T1. Although PLDH1 was able to drive detectable fluorescence expression, its overall activity was lower than that of P32, while PLDH2 showed relatively weak activity. These findings suggest that endogenous promoters derived from the host genome do not necessarily provide optimal expression performance in heterologous systems. Their activity may depend on intrinsic promoter strength, ribosome binding efficiency, transcriptional regulation, and host-specific physiological conditions. In contrast, the P32 promoter maintained strong and stable constitutive activity in *L.c* T1, supporting its application in fluorescent labeling and heterologous expression in lactic acid bacteria.

The choice of fluorescent protein also critically affects imaging performance (Martineau et al., 2009; Salomé-Desnoulez et al., 2021). In this study, mKate consistently exhibited higher fluorescence intensity than mCherry in both *Escherichia coli* DH5α and *L.c* T1. Previous studies have shown that mKate possesses improved photostability and faster chromophore maturation, and longer emission wavelengths, which can reduce interference from tissue autofluorescence during animal imaging (Shcherbo et al., 2007). Accordingly, mKate was selected as a more suitable reporter for gastrointestinal bacterial tracking. Consistently, whole-body fluorescence imaging of mice demonstrated that the P32-mKate strain generated detectable signals after oral administration, supporting its utility for monitoring bacterial distribution *in vivo*.

Notably, fluorescence expression of *L.c* T1-P32-mKate was strongly influenced by environmental pH. Both fluorescence intensity and reporter gene transcription levels were enhanced under mildly alkaline conditions, whereas acidic environments markedly reduced fluorescence output. During growth, lactic acid bacteria continuously produce organic acids, resulting in environmental acidification. Such acidic conditions may influence fluorescent protein folding and chromophore maturation. In addition, pH changes may affect promoter activity and bacterial metabolic states, thereby altering heterologous gene expression. Considering the substantial pH variation along the gastrointestinal tract, environmental conditions may contribute to regional differences in fluorescence intensity observed during bacterial tracking.

Furthermore, fluorescence imaging demonstrated that engineered *L.c* T1 could be detected after oral administration. In the present study, whole-body fluorescence imaging of living mice was performed using a small-animal fluorescence imaging system, which detects emitted fluorescence signals through the intact body surface without surgical intervention. Weak fluorescence signals observed around some non-gastrointestinal anatomical regions, such as the joint areas, were unlikely to represent bacterial localization, but may have resulted from optical effects associated with whole-body imaging, including tissue scattering, signal attenuation, and anatomical variations affecting fluorescence distribution. To further determine the anatomical localization of fluorescent bacteria, gastrointestinal tissues were subsequently collected after euthanasia and subjected to ex vivo fluorescence imaging. These complementary approaches enabled non-invasive monitoring of overall fluorescence signals in living animals while providing tissue-level localization information of engineered *L.c* T1 within the gastrointestinal tract. Compared with wild-type, empty-vector control, and P32-mCherry strains, the P32-mKate strain exhibited stronger and more stable fluorescence signals, supporting its applicability for tracking engineered probiotics in preclinical animal models.

Nevertheless, the current fluorescent reporter system remains primarily applicable to small-animal studies. Although it enables non-invasive visualization of engineered *L.c* T1 in living mice, the use of visible fluorescent proteins such as mKate is limited by tissue penetration depth, signal attenuation, and background autofluorescence, which may restrict direct translation to larger animals or clinical applications. Future development of next-generation tracking strategies, including near-infrared fluorescent reporters, bioluminescence-based systems, or other clinically compatible imaging modalities, may further improve the feasibility of monitoring engineered bacteria in larger biological systems. In addition, this study mainly focused on short-term gastrointestinal tracking after gavage, and the long-term colonization dynamics, persistence, and genetic stability of the engineered strain remain to be systematically investigated. Since the current system relies on plasmid-based expression, potential plasmid loss during prolonged colonization may affect reporter stability. Future studies incorporating chromosomal integration strategies will be valuable for improving long-term expression stability and advancing engineered bacterial tracking applications.

## 4 Conclusion

In this study, we established a red fluorescent reporter platform for *L.c* T1 and identified the P32-mKate combination as an efficient and stable system for non-invasive fluorescence monitoring in living mice. This platform enables visualization of engineered *L.c* T1 distribution within the gastrointestinal tract and provides a valuable tool for investigating probiotic colonization behavior and host–microbe interactions. Although the current fluorescence-based system is primarily applicable to preclinical small-animal models due to limitations in tissue penetration and signal attenuation, it establishes a foundation for future development of advanced tracking strategies and engineered *Lactobacillus* delivery systems.

## 5 Materials and methods

### 5.1 Strains, culture media and conditions

All strains and plasmids used in this study are listed in Supplementary Table S1. *Escherichia coli* DH5α (*E.coli* DH5α) was used exclusively for plasmid construction and preliminary reporter screening. *Lactobacillus casei* T1 (*L.c* T1) was isolated from Tibetan yak milk by our laboratory, and previous studies have demonstrated its potential anti-inflammatory activity and modulatory effects on gut microbiota. *E. coli* DH5α was cultured in Luria–Bertani (LB) medium, while *L.c* T1 was cultured in de Man, Rogosa and Sharpe (MRS) broth. For preparation of electrocompetent *L.c* T1 cells, MRSGS medium supplemented with 4.0% (w/v) glycine and 0.5 mol·L^−1^ sucrose was used. LB medium contained 10 g·L^−1^ tryptone, 5 g·L^−1^ yeast extract, and 10 g·L^−1^ NaCl. MRS medium was purchased from Solarbio (Beijing, China). *E. coli* DH5α was cultured at 37 °C with shaking at 250 rpm. *L.c* T1 was cultured at 37 °C under static conditions. When necessary, antibiotics were added at appropriate concentrations (50 μg·mL^−1^ kanamycin and 20 μg·mL^−1^ erythromycin).

### 5.2 DNA manipulation

Plasmid extraction kits were purchased from Sangon Biotech (Shanghai, China). High-fidelity Phanta Max Super-Fidelity DNA Polymerase, one-step cloning kits, and restriction enzymes were obtained from Vazyme (Nanjing, China) and Thermo Fisher Scientific (Shanghai, China). Primer sequences are listed in Supplementary Table S2. Construction of pMG36E-Px-mKate (*X* = P23, PLDH1, PLDH2) was performed by linearization of pMG36E via inverse PCR, DpnI-mediated template digestion, amplification of mKate from pSD-RFP, seamless recombination, and transformation screening. For PLDH1 and PLDH2 constructs, pMG36E-P32-mKate and corresponding promoter fragments were used as templates. pMG36E-Px-mCherry constructs (*X* = P23, PLDH1, PLDH2) were generated using a similar strategy, with pMG36E-P32-mKate and pUC57-mCherry as templates for plasmid linearization and mCherry amplification. Recombinant plasmids were confirmed by sequencing. Notably, constructs containing PLDH3 (pMG36E-PLDH3-mKate and pMG36E-PLDH3-mCherry) failed to yield positive transformants and were excluded from further experiments.

### 5.3 Preparation of competent cells and electroporation

Electrocompetent *L.c* T1 cells were prepared as follows:

1. A single colony was inoculated into MRS medium supplemented with 5% (w/v) glucose and cultured overnight at 37 °C.
2. The culture was inoculated into 100 mL MRS medium containing 5% glucose and 0–4% glycine and grown at 37 °C until OD_600_ reached 0.2(~2.0 × 10^8^ CFU/mL) – 0.8 (~7.0 × 10^8^ CFU/mL). Cells were then chilled on ice for 30 min and harvested by centrifugation (4 °C, 5000 × g, 5 min).
3. The pellet was washed three times with pre-cooled sterile wash buffer containing 10% (w/v) glucose and 0–4% glycine.
4. Cells were resuspended in 1.0 mL cold wash buffer, and 400 μL aliquots were distributed into electroporation cuvettes.

For electroporation:

1. Plasmid DNA was added and incubated on ice for 30 min.
2. Electroporation was performed at 2.2 kV, 400 *Ω*, with a 0.4 cm cuvette gap. Immediately after pulsing, 1.0 mL pre-warmed MRS medium was added for recovery.
3. Cells were transferred into 5 mL antibiotic-free MRS medium and incubated at 37 °C, 150 rpm for 4 h.
4. Cells were plated on MRS agar containing erythromycin and incubated at 37 °C for 2–3 days.
5. Positive transformants were verified by colony PCR and sequencing.

### 5.4 Measurement of cell growth

Wild-type *L.c* T1, empty vector control (*L.c* T1-pMG36E), and fluorescent strains (*L.c* T1-Px-mKate/mCherry) were inoculated into MRS medium with or without erythromycin and cultured overnight. Cultures were adjusted to OD_600_ = 0.7 (~5.0 × 10^8^ CFU/mL) and incubated at 37 °C in static conditions. OD_600_ was measured at 0, 4, 8, 12, 16, 20, 24, 36, 48, 60, 72, 84, and 96 h using a UV–Vis spectrophotometer. All experiments were performed in triplicate. All experiments were performed using at least three independent biological replicates (*n* = 3).

### 5.5 Confocal laser scanning microscopy

After 24 h culture, cells from wild-type, empty vector, and fluorescent strains were collected by centrifugation. Pellets were washed with PBS and resuspended in 20 μL PBS. A 5 μL aliquot was placed on a glass slide for fluorescence imaging using a confocal laser scanning microscope (excitation: 562 nm).

### 5.6 Fluorescence signals of bacterial cells were captured and quantified using a fluorescence imaging system

Overnight cultures (24 h) of different fluorescently labeled *L.c* T1 strains were collected (5 mL) and resuspended in PBS to normalize the bacterial concentration to an OD_600_ value of 1.4 (~3.0 × 10^9^ CFU/mL). For fluorescence detection [*In vivo* bioluminescence imaging was performed using the NEWTON 7.0 imaging system (Vilber Bio Imaging, France)], 200 μL of the standardized bacterial suspension was added to the first well of an eight-tube strip, while the remaining seven wells were preloaded with 100 μL PBS. A two-fold serial dilution was subsequently performed by transferring 100 μL of bacterial suspension sequentially into the next well, resulting in a dilution series corresponding to 2^0^, 2^1^, 2^2^, 2^3^, 2^4^, 2^5^, and 2^6^, with the final well containing PBS alone as the blank control. The eight-tube strip was placed in an *in vivo* imaging system equipped with a 580 nm excitation wavelength to visualize bacterial fluorescence signals. The acquired images were uniformly adjusted using Kuant software, and fluorescence intensity was quantified by selecting identical regions of interest (ROIs) corresponding to the bacterial suspension area. The relative fluorescence intensity was calculated and plotted to generate fluorescence expression curves for each engineered strain. All experiments were performed using at least three independent biological replicates (*n* = 3).

### 5.7 Fluorescence measurement with a microplate reader (measurement of fluorescence intensity)

Strains were cultured in MRS medium for 24 h, harvested, and resuspended in PBS to OD600 = 1.4 (~3.0 × 10^9^ CFU/mL). A serial dilution was performed in a 96-well plate (final dilutions: 2^0^ to 2^6^ (bacterial count:~3.0 × 10^9^ to 4.7 × 10^7^ CFU/mL), with PBS as blank control. Fluorescence was measured using a microplate reader [Synergy™ H4 Hybrid Multi-Mode Microplate Reader (BioTek Instruments, United States)] under the following conditions: excitation 540/25 nm, emission 620/40 nm, gain 35. All experiments were performed using at least three independent biological replicates (*n* = 3).

### 5.8 Quantitative reverse transcription-PCR

Cells were collected after 24 h culture, washed with PBS, and treated with lysozyme (5 mg/mL) at 37 °C for 30 min. After centrifugation, RNA was extracted using TRIzol reagent following manufacturer instructions. RNA was precipitated with isopropanol, washed with 75% ethanol, and dissolved in RNase-free water. cDNA was synthesized and used for quantitative PCR analysis to assess gene expression levels. All experiments were performed using at least three independent biological replicates (*n* = 3).

### 5.9 Optimization of fluorescent protein expression

According to the growth curve, both wild-type *L.c* T1 and engineered fluorescent strains entered the late exponential phase around 12 h and gradually reached the stationary phase thereafter. The 24 h time point represents the later stationary phase, during which bacterial biomass remains relatively stable and intracellular fluorescent proteins have sufficient time to accumulate. Therefore, these two time points were selected to evaluate fluorescence performance during different stages of bacterial growth while minimizing the influence of rapid changes in cell proliferation. *L.c* T1-P32-mKate and *L.c* T1-P32-mCherry were inoculated into MRS medium and cultured for 24 h. Cultures were then scaled up to 200 mL fresh MRS medium with initial pH adjusted to 6.0, 7.0, or 8.0, and incubated at 37 °C. After 12 h, fluorescence intensity was measured. The pH of all cultures was then readjusted to initial values and incubation continued to 24 h, followed by a second fluorescence measurement. qRT-PCR was performed to assess transcriptional levels at both time points. All experiments were performed using at least three independent biological replicates (*n* = 3).

### 5.10 *In vivo* bioluminescence and fluorescence imaging

Six to eight-week-old female C57BL/6 mice were randomly divided into four groups (*n* = 4 per group): wild-type *L.c* T1, empty vector control (*L.c* T1-pMG36E), *L.c* T1-P32-mKate, and *L.c* T1-P32-mCherry. Each mouse was orally administered 400 μL bacterial suspension (OD_600_ = 2.4, ~4.0 × 10^11^ CFU/mL). In vivo imaging was performed at 0 h and 7 h post-gavage using an *in vivo* imaging system at 580 nm. After 7 h, mice were sacrificed, and the entire gastrointestinal tract (from stomach to anus) was excised. Ex vivo fluorescence imaging was performed under identical conditions. The sample size was determined by a power analysis using GPower software (version 3.1). Fluorescence intensity was selected as the primary outcome parameter. Based on the expected difference in fluorescence intensity between fluorescent and control strains, four mice per group were estimated to provide sufficient statistical power (*α* = 0.05, power = 0.80) for detecting biologically relevant differences.

### 5.11 Statistical analysis

All analyses were performed by comparing experimental groups to their respective controls using the non-parametric one-way analysis of variance Mann–Whitney U-testing or by the two-tailed Student-t testing where appropriate (GraphPad Prism 6.0, California, United States). Data are presented as Means ± SEM. Differences were judged to be statistically significant when the *p*-value was < 0.05. However, *p*-values between 0.05 and 0.1 are specified to indicate trends.

## Data Availability

The raw data supporting the conclusions of this article will be made available by the corresponding authors upon request.
