# Supplementary material for: A fluorescent reporter system for tracking Lactobacillus casei T1 in the murine gastrointestinal tract
Source: Front Microbiol. 2026 Aug 27;17:1892263. doi: 10.3389/fmicb.2026.1892263 (PMC13558207; doi:10.3389/fmicb.2026.1892263)
Supplement: Supplementary file 1 [file Supplementary_file_1.docx]

**Table S1: Strains and plasmids in this study**

| **Strains or plasmids** | **Relevant characteristics** | **References** |
| --- | --- | --- |
| **Strains** |  |  |
| *Escherichia coli* DH5α (DH5α) | Gene cloning strain | Our laboratory |
| *DH5α-pMG36e* | DH5α with pMG36e | This study |
| *DH5α-pMG36e-P32-mKate* | DH5α with pMG36e-P32-mKate | This study |
| *DH5α-pMG36e-PLDH1-mKate* | DH5α with pMG36e-PLDH1-mKate | This study |
| *DH5α-pMG36e-PLDH2-mKate* | DH5α with pMG36e-PLDH2-mKate | This study |
| *DH5α-pMG36e-P32-mCherry* | DH5α with pMG36e-P32-mCherry | This study |
| *DH5α-pMG36e-PLDH1-mCherry* | DH5α with pMG36e-PLDH1-mCherry | This study |
| *DH5α-pMG36e-PLDH2-mCherry* | DH5α with pMG36e-PLDH2-mCherry | This study |
| *Lactobacillus casei* T1 (*L.c* T1) | Wild type strain | (Wu et al., 2021) |
| *L.c* T1*-pMG36e* | Derived from *L.c* T1, containing plasmid pMG36e | This study |
| *L.c* T1*-pMG36e-P32-mKate* | Derived from *L.c* T1, containing plasmid pMG36e-P32-mKate | This study |
| *L.c* T1*-pMG36e-PLDH1-mKate* | Derived from *L.c* T1, containing plasmid pMG36e-PLDH1-mKate | This study |
| *L.c* T1*-pMG36e-PLDH2-mKate* | Derived from *L.c* T1, containing plasmid pMG36e-PLDH2-mKate | This study |
| *L.c* T1*-pMG36e-P32-mCherry* | Derived from *L.c* T1, containing plasmid pMG36e-P32-mCherry | This study |
| *L.c* T1*-pMG36e-PLDH1-mCherry* | Derived from *L.c* T1, containing plasmsid pMG36e-PLDH1-mCherry | This study |
| *L.c* T1*-pMG36e-PLDH2-mCherry* | Derived from *L.c* T1, containing plasmsid pMG36e-PLDH2-mCherry | This study |
| **Plasmids** |  |  |
| pSD-RFP | Kan*, containing *mKate* gene | Our laboratory |
| pUC57-mCherry | Amp*, containing *mCherry* gene | Our laboratory |
| pMG36e | Erm*; expression vector with the *P32* promoter, multiple cloning sites (MCFs), and *prtP* translational terminator | Our laboratory |
| pMG36e-P32-mKate | Derived from pMG36e, containing  P32-mKate expression box | This study |
| pMG36e-PLDH1-mKate | Derived from pMG36e, containing  PLDH1-mKate expression box | This study |
| pMG36e-PLDH2-mKate | Derived from pMG36e, containing  PLDH2-mKate expression box | This study |
| pMG36e-P32-mCherry | Derived from pMG36e-P32-mKate, containing P32-mCherry expression box | This study |
| pMG36e-PLDH1-mCherry | Derived from pMG36e-P32-mKate, containing PLDH1-mCherry expression box | This study |
| pMG36e-PLDH2-mCherry | Derived from pMG36e-P32-mKate, containing PLDH2-mCherry expression box | This study |

**Table S2: Primers information in this study**

| **Primers** | **Sequences (5′ to 3′)** | **Note** |
| --- | --- | --- |
| pMG36e-P32-mKate-1 | taattctgacatttcaaaattcctccgaatatttttttacc | Linearized pMG36e |
| pMG36e-P32-mKate-2 | catcgttgattgaaccttgcttaggcagctgacttc |  |
| pMG36e-P32-mKate-3 | gaattttgaaatgtcagaattaattaaagaaaatatgc | mKate fragment was amplified from pSD-RFP |
| pMG36e-P32-mKate-4 | ctaagcaaggttcaatcaacgatgtcctaatttcgacgga |  |
| pMG36e-PLDH1-mKate-1 | tggtagctttttaaatatgggtcgatatctgatagagatgtcatgaag | Linearized pMG36e-P32-mKate |
| pMG36e-PLDH1-mKate-2 | tcatgtgcatattttctttaattaattctgacatgaaaacccttcccctccactcc |  |
| pMG36e-PLDH1-mKate-3 | atctctatcagatatcgacccatatttaaaaagcta | PLDH1 fragment was amplified from the genome of *L.c* T1. |
| pMG36e-PLDH1-mKate-4 | gaagggttttcatgtcagaattaattaaagaaaatatgcac |  |
| pMG36e-PLDH2-mKate-1 | aggaggataaaagttccatcgacccatatttaaaaagc | Linearized pMG36e-P32-mKate |
| pMG36e-PLDH2-mKate-2 | aaggatgatatcaccatgtcagaattaattaaagaaaatatgc |  |
| pMG36e-PLDH2-mKate-3 | atgggtcgatggaacttttatcctccttg | PLDH2 fragment was amplified from the genome of *L.c* T1. |
| pMG36e-PLDH2-mKate-4 | ttaattaattctgacatggtgatatcatcctttcttatg |  |
| pMG36e-P32-mCherry-1 | cttgctcaccatttcaaaattcctccgaatatttttttacc | Linearized pMG36e |
| pMG36e-P32-mCherry-2 | atggacgagctgtacaagtgattgaaccttgcttaggcagctgac |  |
| pMG36e-P32-mCherry-3 | ggaggaattttgaaatggtgagcaagggcgagg | mCherry fragment was amplified from pUC57-mCherry |
| pMG36e-P32-mCherry-4 | ggttcaatcacttgtacagctcgtccatgccg |  |
| pMG36e-PLDH1- mCherry-1 | caagtaattgaaccttgcttaggcagctg | Linearized pMG36e-PLDH1-mKate |
| pMG36e-PLDH1- mCherry-2 | accatggtgatatcatcctttcttatgtgcatgc |  |
| pMG36e-PLDH1-mCherry-3 | taagaaaggatgatatcaccatggtatctaagggtgaggagg | mCherry fragment was amplified from pUC57-mCherry |
| pMG36e-PLDH1- mCherry-4 | tcagctgcctaagcaaggttcaattacttgtataattcgtccatccc |  |
| pMG36e-PLDH2- mCherry-1 | acccttagataccatctcaaattcctcctcatgaagatcttg | Linearized pMG36e-PLDH2-mKate |
| pMG36e-PLDH2- mCherry-2 | cgaattatacaagtaattgaaccttgcttaggcagctg |  |
| pMG36e-PLDH2- mCherry-3 | atgaggaggaatttgagatggtatctaagggtgaggag | mCherry fragment was amplified from pUC57-mCherry |
| pMG36e-PLDH2- mCherry-4 | ctgcctaagcaaggttcaattacttgtataattcgtccatccc |  |
| pMG36e-Clone-test-1 | catcctcttcgtcttggt | Primer for Recombinant Vector Detection |
| pMG36e-Clone-test-2 | gcttgtttgattttcaaacttc |  |
| mCherry-1 | ggctattatcaaagaatttatgcg | Primer for *mCherry* detection |
| mCherry-2 | cttttgtaacttttaacttagcg |  |
| mKate-1 | gaattttgaaatgtcagaattaattaaagaaaatatgc | Primer for *mKate* detection |
| mKate-2 | ctaagcaaggttcaatcaacgatgtcctaatttcgacgga |  |

**Table S3 Determination of final pH values of fluorescent strains after 12 h and 24 h of incubation**

|  | 12h | | | | 24h | | | |
| --- | --- | --- | --- | --- | --- | --- | --- | --- |
|  | ① | ② | ③ | Mean ± SD | ① | ② | ③ | Mean ± SD |
| pH_6.0_ | 4.50 | 4.55 | 4.33 | 4.46 ± 0.12 | 4.42 | 4.59 | 4.44 | 4.48 ± 0.09 |
| pH_7.0_ | 4.51 | 4.43 | 4.58 | 4.51 ± 0.08 | 4.57 | 4.50 | 4.52 | 4.53 ± 0.04 |
| pH_8.0_ | 4.49 | 4.71 | 4.73 | 4.64 ± 0.13 | 4.61 | 4.66 | 4.83 | 4.70 ± 0.12 |

**Table S4 Viable cell counting of fluorescent strains after incubation at different pH values at various time points**

| pH = 6 | 12h | | | 24h | | |
| --- | --- | --- | --- | --- | --- | --- |
|  | Conunt. 1 | Conunt. 2 | Conunt. 3 | Conunt. 1 | Conunt. 2 | Conunt. 3 |
| *L.c* T1-pMG36e | 591 | 632 | 604 | 592.00 | 740 | 850 |
| *L.c* T1-p32-mKate | 234 | 406 | 124 | 88 | 91 | 85 |
| pH = 7 |  |  |  |  |  |  |
|  | Conunt. 1 | Conunt. 2 | Conunt. 3 | Conunt. 1 | Conunt. 2 | Conunt. 3 |
| *L.c* T1-pMG36e | 562 | 542.00 | 577 | 991 | 884 | 776 |
| *L.c* T1-p32-mKate | 76 | 62 | 65 | 725 | 1413 | 707 |
| pH = 8 |  |  |  |  |  |  |
|  | Conunt. 1 | Conunt. 2 | Conunt. 3 | Conunt. 1 | Conunt. 2 | Conunt. 3 |
| *L.c* T1-pMG36e | 552 | 603 | 628 | 1140 | 1191 | 1029 |
| *L.c* T1-p32-mKate | 476 | 602 | 584 | 910 | 727 | 757 |
